# Treatment and Survival of Elderly Patients with Stage I–II Pancreatic Cancer: A Report of the EURECCA Pancreas Consortium

**DOI:** 10.1245/s10434-020-08539-x

**Published:** 2020-05-09

**Authors:** Jesse V. Groen, Tom A. Douwes, Elizabeth van Eycken, Lydia G. M. van der Geest, Tom B. Johannesen, Marc G. Besselink, Bas Groot Koerkamp, Johanna W. Wilmink, Bert A. Bonsing, Johanna E. A. Portielje, Cornelus J. H. van de Velde, Esther Bastiaannet, J. Sven D. Mieog, on behalf of the Dutch Pancreatic Cancer Group

**Affiliations:** 1 Department of Surgery, Leiden University Medical Center, Albinusdreef 2, 2300 RC Leiden, The Netherlands; 2 Belgian Cancer Registry, Brussels, Belgium; 3 Department of Research and Development, Netherlands Comprehensive Cancer Organisation (IKNL), Utrecht, The Netherlands; 4 Registry Department, The Cancer Registry of Norway, Oslo, Norway; 5 Department of Surgery, Cancer Center Amsterdam Amsterdam, UMC, University of Amsterdam, Amsterdam, The Netherlands; 6 Department of Surgery, Erasmus Medical Center, Rotterdam, The Netherlands; 7 Department of Medical Oncology, Cancer Center Amsterdam, UMC, University of Amsterdam, Amsterdam, The Netherlands; 8 Department of Medical Oncology, Leiden University Medical Center, Leiden, The Netherlands

## Abstract

**Background:**

Elderly patients with pancreatic cancer are underrepresented in clinical trials, resulting in a lack of evidence.

**Objective:**

The aim of this study was to compare treatment and overall survival (OS) of patients aged ≥ 70 years with stage I–II pancreatic cancer in the EURECCA Pancreas Consortium.

**Methods:**

This was an observational cohort study of the Belgian (BE), Dutch (NL), and Norwegian (NOR) cancer registries. The primary outcome was OS, while secondary outcomes were resection, 90-day mortality after resection, and (neo)adjuvant and palliative chemotherapy.

**Results:**

In total, 3624 patients were included. Resection (BE: 50.2%; NL: 36.2%; NOR: 41.3%; *p* < 0.001), use of (neo)adjuvant chemotherapy (BE: 55.9%; NL: 41.9%; NOR: 13.8%; *p* < 0.001), palliative chemotherapy (BE: 39.5%; NL: 6.0%; NOR: 15.7%; *p* < 0.001), and 90-day mortality differed (BE: 11.7%; NL: 8.0%; NOR: 5.2%; *p* < 0.001). Furthermore, median OS in patients with (BE: 17.4; NL: 15.9; NOR: 25.4 months; *p* < 0.001) and without resection (BE: 7.0; NL: 3.9; NOR: 6.5 months; *p* < 0.001) also differed.

**Conclusions:**

Differences were observed in treatment and OS in patients aged ≥ 70 years with stage I–II pancreatic cancer, between the population-based cancer registries. Future studies should focus on selection criteria for (non)surgical treatment in older patients so that clinicians can tailor treatment.

**Electronic supplementary material:**

The online version of this article (10.1245/s10434-020-08539-x) contains supplementary material, which is available to authorized users.

For pancreatic cancer, very little progress has been made in terms of mortality rates over the past decades.^1^ Resection combined with systemic treatment offers the best chance for prolonged survival. Resectability is mainly determined by contact between the tumor and the venous and arterial vasculature.^2^ Patients with stage I–II pancreatic cancer are generally considered eligible for resection. Unfortunately, about 20% of all patients are resectable due to advanced or metastatic disease at diagnosis.^3^ Still, even after tumor resection of stage I–II pancreatic cancer, prognosis is poor, with a median overall survival (OS) of 17–30 months.^4^

The most recent European Society of Medical Oncology (ESMO) guideline does not consider advanced age a contraindication for resection, but states that comorbidities and poor functional status can be a reason to refrain from resection.^5^ The National Comprehensive Cancer Network (NCCN) guideline is largely similar to the ESMO guideline.^6^ Although no statements are made regarding advanced age directly, the guideline states that performance status should be taken into account when considering treatment strategy. Older cancer patients are often underrepresented in clinical trials, possibly due to the strict inclusion criteria.^7^ Recently, a study with population-based data of multiple pancreatic cancer registries showed that the median age at diagnosis is 70 years.^8^ This clearly differs from large randomized controlled trials in pancreatic cancer in which the median age is 61–65 years.^9^–^12^ There is a lack of evidence on treatment and survival of elderly patients with pancreatic cancer.

The EUropean REgistration of Cancer CAre (EURECCA) consortium, established by the European CanCer Organisation (ECCO), investigates differences in treatment and outcomes of patients in a real-world scenario by using cancer registry data.^13^ Previous studies from the EURECCA Pancreas Consortium showed considerable variations in treatment and outcomes.^14^,^15^

The aim of this study was to compare treatment strategies and survival outcomes of patients aged ≥ 70 years with stage I–II pancreatic cancer in the Belgian (BE), Dutch (NL), and Norwegian (NOR) national cancer registries from the EURECCA Pancreas Consortium.

## Methods

### Design and Patient Selection

This was an observational cohort study of three cancer registries in the EURECCA Pancreas Consortium reported according to the Strengthening the Reporting of Observational Studies (STROBE) criteria.^16^ The BE, NL, and NOR national cancer registries were selected because of data quality, data availability, and similarity regarding design and organization (electronic supplementary Table S1). In addition, cancer incidence and life expectancy are largely similar between these national cancer registries.^17^ Patients aged ≥ 70 years with pancreatic adenocarcinoma stage I–II, diagnosed from 2012 through 2016 (2012 through 2015 for BE), were included. Patients aged ≥ 70 years were included according to the definitions of ‘elderly’ of the International Society of Geriatric Oncology (http://siog.org/content/defining-elderly). An overview of stage distribution per cancer registry is provided in electronic supplementary Table S2. Patients with other malignancies were not excluded because pancreatic cancer is often determinative for the prognosis. In case of synchronous pancreatic cancer, the tumor with the highest known stage was used.

### Data Collection, Definition, and Preparation

Anonymous data obtained from the cancer registries included (1) patient- and tumor-related variables, i.e. sex, age, tumor topography, tumor morphology, tumor stage; (2) treatment-related variables, i.e. tumor resection, chemotherapy, radiotherapy; and (3) outcome-related variables, i.e. vital status, follow-up.

Patients were divided into three age groups: 70–74, 75–79, and ≥ 80 years. The International Classification of Diseases for Oncology, Third Revision (ICD-O-3) was used for tumor topography and morphology.^18^ Pancreatic cancers were identified through tumor topography codes (C25.0, C25.1, C25.2, C25.3, C25.7, C25.8, C25.9) and morphological codes (8000–8009, 8010–8012, 8014–8049, 8050–8089, 8140–8149, 8154, 8158, 8159, 8161, 8163–8169, 8171–8179, 8181–8239, 8244–8245, 8250–8311, 8313–8389, 8440–8499, 8500–8549, 8550–8559, 8560–8579). For NOR, morphological codes 690099 and 699999 (no or unknown microscopic examination) were also included, since similar patients are coded as 8000 in the BE and NL cancer registries. Unless patients with codes 690099 and 699999 were diagnosed by death certificate only, these patients are not included in the BE and NL cancer registries.

The 7th edition of the TNM classification was in use during the study period and was therefore used for tumor staging in BE and NL.^19^ pTNM stage was used in patients who underwent tumor resection and cTNM stage was used in patients who did not undergo tumor resection. In case of missing pTNM stage variables for patients who underwent tumor resection, cTNM stage variables were used when available. In NOR, tumor stage was categorized as localized, regional, or distant disease. For analyses, localized and regional tumor disease were included. In case of missing data on tumor resection, chemotherapy, and radiotherapy, these categories were classified as ‘no’. No distinction was made between neo- and adjuvant nonsurgical treatment since these data were not available for NOR. OS was calculated from the day of diagnosis or tumor resection until the date of death or last follow-up.

### Outcomes and Comparisons

The primary outcome was OS, while secondary outcomes were tumor resection, 90-day mortality after tumor resection, and use of nonsurgical treatment strategies [(neo)adjuvant and palliative chemotherapy and radiotherapy]. The main comparison focused on assessing differences in the three cancer registries. Subgroup analyses were performed comparing each age group between the cancer registries (in cases of ≥ 60 events).

### Statistical Analyses

Statistical analyses were performed using SPSS Inc. for Windows version 23.0 (IBM Corporation, Armonk, NY, USA). Categorical data were reported as numbers (percentages) and were compared using the Chi square test. Multivariable binary logistics regression was used to assess predictive factors (cancer registry, age group) for tumor resection and 90-day mortality after tumor resection, as well as use of nonsurgical treatment strategies [(neo)adjuvant and palliative chemotherapy and radiotherapy] (in cases of ≥ 60 events). Survival analyses were performed separately for patients who underwent tumor resection and patients who did not undergo tumor resection. Kaplan–Meier curves were used to estimate the median OS and 95% confidence interval (CI), and log-rank tests were used to compare OS. Multivariable Cox regression was used to assess predictive factors (cancer registry, age group) for OS. BE and age group 70–74 years were the reference categories in the multivariable analyses. Sensitivity analyses were performed, excluding patients who deceased within 90 days after tumor resection or diagnosis, and including chemotherapy as an additional factor to assess the influence on OS and minimize confounding by indication. In patients who did not undergo tumor resection, a sensitivity analysis was performed only for patients in which the tumor was pathologically confirmed. The original results were considered robust if the sensitivity analyses showed similar results. A *p* value < 0.05 was considered statistically significant for all analyses.

## Results

### Patient and Tumor Characteristics

In total, 3624 patients were included: 1002 (27.6%) from BE, 1973 (54.4%) from NL, and 649 (17.9%) from NOR (Table 1). Distribution of sex was comparable between the cancer registries, and age group distribution was largely similar. Most tumors were stage II/regional (72.1% in BE; 67.4% in NL; 72.0% in NOR).

**Table 1:** Patient and tumor characteristics by cancer registry

		Cancer registry					
		BE		NL		NOR	
		*n*	%	*n*	%	*n*	%
Total		1002	27.6	1973	54.4	649	17.9
Age group, years	70–74	300	29.9	545	27.6	216	33.3
	75–79	310	30.9	564	28.6	166	25.6
	≥80	392	39.1	864	43.8	267	41.1
Sex	Male	458	45.7	894	45.3	295	45.5
	Female	544	54.3	1079	54.7	354	54.5
Stage^a^	IA	79	7.9	158	8.0	182	28.0
	IB	201	20.1	485	24.6		
	IIA	226	22.6	552	28.0	467	72.0
	IIB	496	49.5	778	39.4		

*BE* Belgian, *NL* Dutch, *NOR* Norwegian

^a^ For NOR, no distinction was made for stage IA/IB and IIA/IIB

### Treatment Strategies

#### Tumor Resection

**Fig. 1:**
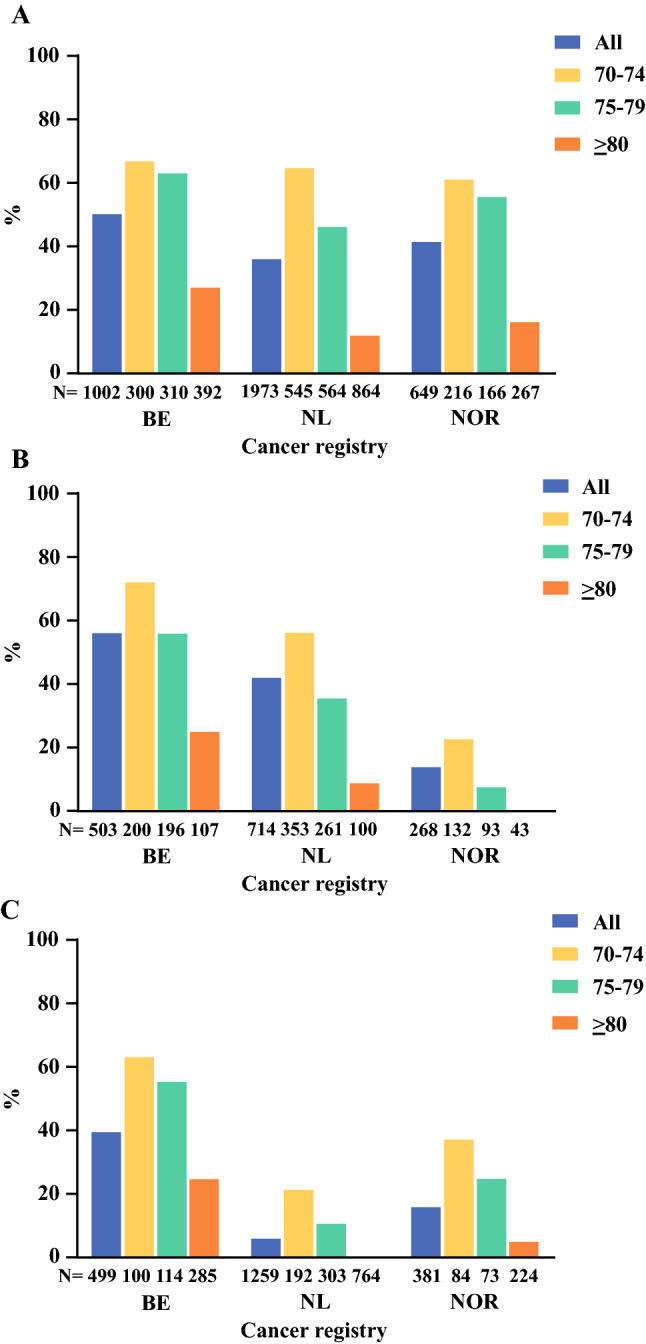
Treatment strategies for (**a**) tumor resection, (**b**) (neo)adjuvant chemotherapy, and (**c**) palliative chemotherapy, by cancer registry and age group. *BE* Belgian, *NL* Dutch, *NOR* Norwegian

The tumor resection rate differed between the cancer registries: 50.2% in BE, 36.2% in NL, and 41.3% in NOR (*p* < 0.001) [Fig. 1a]. Subgroup analysis showed a similar tumor resection rate in the 70–74 years age group (*p* = 0.424) and different tumor resection rates in the higher age groups between the registries (both *p* < 0.001).

In multivariable analyses, patients in NL (odds ratio [OR] 0.54, 95% CI 0.46–0.65) and NOR (OR 0.65, 95% CI 0.52–0.81) were less likely to undergo tumor resection compared with BE (Table 2). Patients in the 75–79 years (OR 0.61, 95% CI 0.51–0.73) and ≥ 80 years age groups (OR 0.10, 95% CI 0.09–0.13) were less likely to undergo tumor resection compared with the 70–74 years age group.

**Table 2:** Multivariable analyses for treatment strategies

		Tumor resection^a^		(Neo)adjuvant chemotherapy^b^		Palliative chemotherapy^c^	
		OR (95% CI)	*p* value	OR (95% CI)	*p* value	OR (95% CI)	*p* value
Cancer	BE	Reference		Reference		Reference	
Registry	NL	0.54 (0.46–0.65)	<0.001	0.43 (0.34–0.56)	<0.001	0.08 (0.05–0.10)	<0.001
	NOR	0.65 (0.52–0.81)	<0.001	0.09 (0.06–0.13)	<0.001	0.22 (0.15–0.32)	<0.001
Age group, years	70–74	Reference		Reference		Reference	
	75–79	0.61 (0.51–0.73)	<0.001	0.43 (0.34–0.55)	<0.001	0.54 (0.38–0.75)	<0.001
	≥80	0.10 (0.09–0.13)	<0.001	0.10 (0.07–0.15)	<0.001	0.10 (0.07–0.14)	<0.001

#### Nonsurgical Treatment in Patients Who Underwent Tumor Resection

The use of (neo)adjuvant chemotherapy differed between the cancer registries: 55.9% in BE, 41.9% in NL, and 13.8% in NOR (*p* < 0.001) [Fig. 1b]. Subgroup analysis showed that in all age groups, the use of (neo)adjuvant chemotherapy differed between the cancer registries (all *p* < 0.001). In multivariable analyses, patients in NL (OR 0.43, 95% CI 0.34–0.56) and NOR (OR 0.09, 95% CI 0.06–0.13) were less likely to receive (neo)adjuvant chemotherapy compared with BE (Table 2). Patients in the 75–79 years (OR 0.43, 95% CI 0.34–0.55) and ≥ 80 years age groups (OR 0.10, 95% CI 0.07–0.14) were less likely to receive (neo)adjuvant chemotherapy compared with the 70–74 years age group.

The use of (neo)adjuvant radiotherapy was similar between the cancer registries: 4.0% in BE, 2.2% in NL, and 3.7% in NOR (*p* = 0.183).

#### Nonsurgical Treatment in Patients Who Did Not Undergo Tumor Resection

The use of palliative chemotherapy differed between the cancer registries: 39.5% in BE, 6.0% in NL, and 15.7% in NOR (*p* < 0.001) [Fig. 1c]. Subgroup analysis showed that in all age groups, the use of palliative chemotherapy differed between the cancer registries (all *p* < 0.001). In multivariable analyses, patients in NL (OR 0.08, 95% CI 0.05–0.10) and NOR (OR 0.22, 95% CI 0.15–0.32) were less likely to receive palliative chemotherapy compared with BE (Table 2). Patients in the 75–79 years (OR 0.54, 95% CI 0.38–0.75) and ≥ 80 years age groups (OR 0.10, 95% CI 0.07–0.15) were less likely to receive palliative chemotherapy compared with patients in the 70–74 years age group.

The use of palliative radiotherapy differed between the cancer registries: 7.4% in BE, 1.6% in NL, and 0.7% in NOR (*p* < 0.001).

### Survival

#### Ninety-Day Mortality After Tumor Resection

**Fig. 2:**
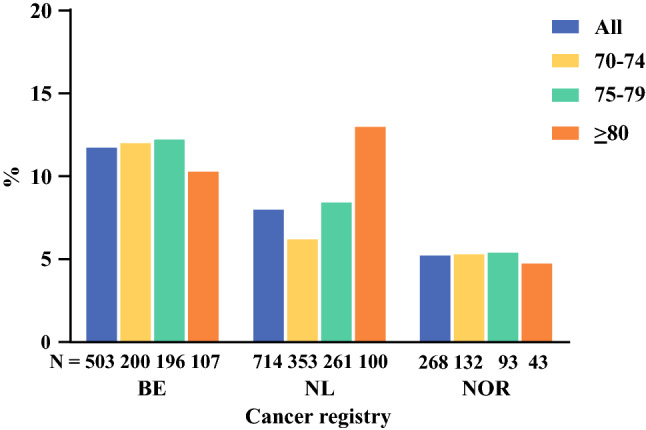
Ninety-day mortality after tumor resection, by cancer registry and age group. *BE* Belgian, *NL* Dutch, *NOR* Norwegian

Ninety-day mortality after tumor resection differed between the cancer registries: 11.7% in BE, 8.0% in NL, and 5.2% in NOR (*p* < 0.001) [Fig. 2]. Subgroup analysis showed different 90-day mortality after tumor resection in the 70–74 years age group (*p* = 0.012), and a similar 90-day mortality after tumor resection in the 75–79 years (*p* = 0.138) and ≥ 80 years age groups (*p* = 0.324) between the cancer registries. In multivariable analyses, patients in NL (OR 0.64, 95% CI 0.43–0.95) and NOR (OR 0.38, 95% CI 0.20–0.72) were less likely to experience 90-day mortality after tumor resection compared with BE (Table 3). Age group was not a significant predictive factor for 90-day mortality after tumor resection.

**Table 3:** Multivariable analyses for survival

		90-day mortality after tumor resection^a^		Overall survival of patients who underwent tumor resection^b^		Overall survival of patients who did not undergo tumor resection^c^	
		OR (95% CI)	*p* value	HR (95% CI)	*p* value	HR (95% CI)	*p* value
Cancer	BE	Reference		Reference		Reference	
Registry	NL	0.67 (0.45–0.98)	0.040	1.07 (0.93–1.22)	0.340	1.46 (1.31–1.62)	<0.001
	NOR	0.42 (0.23–0.77)	0.005	0.72 (0.60–0.87)	0.001	1.35 (1.18–1.55)	<0.001
Age group, years	70–74	Reference		Reference		Reference	
	75–79	1.18 (0.79–1.76)	0.433	1.23 (1.07–1.40)	0.001	1.12 (0.97–1.29)	0.111
	≥80	1.30 (0.79–2.13)	0.307	1.30 (1.10–1.54)	0.002	1.28 (1.14–1.44)	<0.001

*OR* odds ratio, *CI* confidence interval, *HR* hazard ratio, *BE* Belgian, *NL* Dutch, *NOR* Norwegian

^a^Ninety-day mortality in patients who underwent tumor resection (*n* = 1485)

^b^Overall survival of patients who underwent tumor resection (*n* = 1485)

^c^Overall survival of patients who did not undergo tumor resection (*n* = 2139)

#### Overall Survival of Patients Who Underwent Tumor Resection

**Fig. 3:**
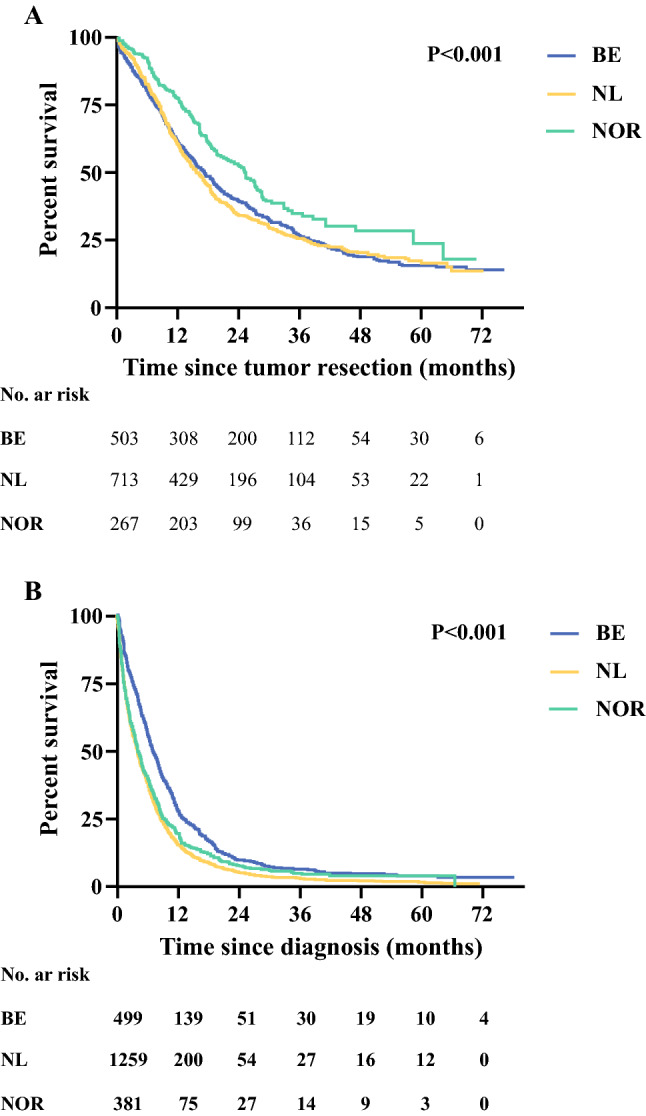
Overall survival by cancer registry for (**a**) patients who underwent tumor resection, and (**b**) patients who did not undergo tumor resection. *BE* Belgian, *NL* Dutch, *NOR* Norwegian

Median OS in patients who underwent tumor resection differed between the cancer registries: 17.4 months (15.3–19.4) in BE, 15.9 months (14.4–17.5) in NL, and 25.4 months (21.6–29.2) in NOR (*p* < 0.001) [Fig. 3a]. Subgroup analysis showed different OS in the 70–74 years age group between the cancer registries, and similar OS in the 75–79 years and ≥ 80 years age groups (electronic supplementary Figs. S1a–c). In multivariable analyses, patients in NL showed similar OS (hazard ratio [HR] 1.07, 95% CI 0.93–1.22) and patients in NOR showed better OS (HR 0.72, 95% CI 0.60–0.87) compared with BE (Table 3). Patients in the 75–79 years (HR 1.23, 95% CI 1.07–1.40) and ≥ 80 years age groups (HR 1.30, 95% CI 1.10–1.54) showed worse OS compared with the 70–74 years age group.

In the sensitivity analysis without patients who deceased within 90 days after tumor resection, patients who received (neo)adjuvant chemotherapy showed better OS compared with (neo)adjuvant chemotherapy-naïve patients; the results according to cancer registry and age group were robust (Table 4 and electronic supplementary Table S3). Detailed analyses by cancer registry and age group showed inconsistent results regarding OS of patients who received (neo)adjuvant chemotherapy versus (neo)adjuvant chemotherapy-naïve patients (electronic supplementary Table S4).

**Table 4:** Sensitivity analyses for overall survival, excluding patients who deceased within 90 days after diagnosis or tumor resection, by age group and treatment strategy

				Age group, years								
Treatment strategy	Total			70–74			75–79			≥80		
	*n*	%	OS (95% CI)^a^	*n*	%	OS (95% CI)^a^	*n*	%	OS (95% CI)^a^	*n*	%	OS (95% CI)^a^
Tumor resection + (neo)adjuvant chemotherapy	602	23.2	22 (19–25)	366	41.6	24 (20–28)	200	24.8	20 (18–23)	36	3.9	21 (13–30)
Tumor resection alone	752	28.9	18 (17–20)	266	30.3	22 (18–26)	298	37.0	16 (14–18)	188	20.5	17 (15–19)
Palliative chemotherapy	293	11.3	9 (8–11)	118	13.4	11 (9–13)	101	12.5	7 (2–12)	74	8.1	10 (8–11)
No treatment	951	36.6	8 (7–9)	129	14.7	12 (10–13)	205	25.5	8 (7–9)	617	67.4	8 (7–9)
Total	2599	100	13 (12–14)	879	100	18 (17–20)	805	100	14 (12–15)	915	100	10 (9–10)

*OR* odds ratio, *CI* confidence interval

^a^Median overall survival (in months) after tumor resection (patients who underwent tumor resection) or after diagnosis (patients who did not undergo tumor resection) and 95% CI

#### Overall Survival of Patients Who Did Not Undergo Tumor Resection

Median OS in patients who did not undergo tumor resection differed between the cancer registries: 7.0 months (6.2–7.8) in BE, 3.9 months (3.5–4.3) in NL, and 6.5 months (5.0–8.0) in NOR (*p* < 0.001) [Fig. 3b]. Subgroup analysis showed different OS in all age groups between the cancer registries (electronic supplementary Figs. S2a–c). In multivariable analyses, patients in NL (HR 1.46, 95% CI 1.31–1.62) and NOR (HR 1.35, 95% CI 1.18–1.55) showed worse OS compared with BE (Table 3), while patients in the 75–79 years ago group showed similar OS (HR 1.12, 95% CI 0.97–1.29) and patients in the ≥ 80 years age group showed worse OS (HR 1.28, 95% CI 1.14–1.44) compared with the 70–74 years age group.

In the sensitivity analysis without patients who deceased within 90 days after diagnosis, patients who received palliative chemotherapy did not show better OS compared with palliative chemotherapy-naïve patients; the results according to cancer registry and age group were robust (Table 4 and electronic supplementary Table S3). Detailed analyses by cancer registry and age group showed inconsistent results regarding the OS of patients who received palliative chemotherapy versus palliative chemotherapy-naïve patients (electronic supplementary Table S4). In the sensitivity analysis including patients in which the tumor was pathologically confirmed, results regarding cancer registries, age group, and palliative chemotherapy were robust.

## Discussion

In this study, the treatment and survival of patients aged ≥ 70 years with stage I–II pancreatic cancer were evaluated in three European population-based cancer registries. Variations were observed for tumor resection rate (range 36–50%), (neo)adjuvant chemotherapy (range 14–56%), and palliative chemotherapy (range 6–40%). Subgroup analysis showed that patients in the 70–74 years age group had a similar tumor resection rate between the cancer registries, which was different in the older age groups. The use of (neo)adjuvant and palliative chemotherapy was different in all age groups between the cancer registries. The use of (neo)adjuvant and palliative radiotherapy was low. Ninety-day mortality after tumor resection was lower in NL and NOR compared with BE. In patients who underwent tumor resection, OS in NOR was better compared with BE, while NL was similar to BE. Overall, an improved OS was observed in patients who received (neo)adjuvant compared with chemotherapy-naïve patients. In patients who did not undergo tumor resection, OS in BE was better compared with NL and NOR.

Although the TNM staging system is not directly translatable to widely used resectability criteria,^5^ the low resection rate in this study, compared with that previously reported,^20^ is noteworthy and could be explained by the inclusion of patients ≥ 70 years of age. In addition, some patients may have anatomically resectable disease, yet have unfavorable biological (high CA19.9) and conditional (poor functional status) factors.^21^ An important observation is that only in the 70–74 years age group was the tumor resection rate similar between the cancer registries. According to the ESMO and NCCN guidelines, poor functional status, but not advanced age, can be a good reason to be more retained by clinician and patients in their choice which treatment is most suitable;^5^,^6^ however, unfortunately, no data (e.g. American Society of Anesthesiologists [ASA], Eastern Cooperative Oncology Group [ECOG] score) were available to investigate this. Variation between the cancer registries regarding the cultural factors that influence the decision making for treatment in elderly patients might also be an explanation.^22^,^23^ Despite the higher tumor resection rates in BE and NOR in the older age groups, which could have illustrated poor patient selection, 90-day mortality after resection was similar. In NL only, 90-day mortality after resection increased with ascending age groups. Possibly, the transparent outcome indicators (mortality) in the Dutch Pancreatic Cancer Audit,^24^ refrains clinicians in NL from performing more tumor resections. A recent meta-analysis showed elderly patients have more comorbidities and more overall complications (mainly respiratory), but comparable mortality compared with younger patients.^25^ Adequate patient selection, prehabilitation, enhanced recovery protocols, and centralization of pancreatic surgery for elderly patients might improve outcomes.^26^–^30^ Others have advocated a multidisciplinary approach to high-risk elderly patients undergoing major surgery,^31^ and several studies have illuminated the importance of geriatric assessment to improve the outcomes of cancer treatment.^32^,^33^ However, high-level evidence regarding functional recovery of elderly patients undergoing pancreatic surgery is lacking. Surprisingly, in a Canadian population-based cohort study,^34^ age was not a predictive factor for functional recovery.

The use of (neo)adjuvant chemotherapy was different between the cancer registries, comparable with previous international studies.^8^,^15^ Nonetheless, this is notable since adjuvant chemotherapy is the standard treatment.^5^,^6^ Morbidity after surgery is not uncommon in elderly patients and may cause omission of chemotherapy.^25^,^26^,^35^ Unfortunately, these data were not available in the present study. No distinction was made between neo- and adjuvant chemotherapy because NOR did not provide this. This was accepted since the use of neoadjuvant therapy was expected to be low as the ESMO and NCCN guidelines stated that neoadjuvant therapy should be used in clinical trials, and that elderly patients are often not included. The sensitivity analyses showed that the differences between the cancer registries regarding OS after tumor resection cannot be explained by the differences in the use of (neo)adjuvant chemotherapy. It remains unknown which other factors also contribute to the differences in OS.

The largest observed difference was in the use of palliative chemotherapy between BE (40%) and NL (6%). This can be explained by the fact that the ESMO and NCCN guidelines state that palliative treatment can be considered depending on the performance status of the patient.^5^ Differences can also be explained by variations in nihilistic attitudes of clinicians and patients regarding the small benefit of palliative chemotherapy in elderly pancreatic cancer patients.^36^ Multiple randomized controlled trials showed improved OS and quality of life with palliative chemotherapy, but adverse events are not rare.^9^,^10^ Exemplified by the present study, results from randomized controlled trials cannot directly be extrapolated to the elderly population due to the strict inclusion criteria. These factors should be discussed with the patient before a shared decision on treatment strategy can be made. In the sensitivity analyses, patients from BE had an improved OS compared with NL, and similar to NOR, which suggests that the differences in the use of palliative chemotherapy do not explain the observed differences in OS. Furthermore, in sensitivity analyses, palliative chemotherapy was not a significant predictive factor for OS. The unclear pattern between (neo)adjuvant and palliative chemotherapy and OS in subgroup analyses suggests that better patient selection is needed to improve resource utilization and OS. However, the results also show that tumor resection and (neo)adjuvant and palliative chemotherapy, in correctly selected patients, can prolong survival.

This study has several limitations. First, although the design and organization of the national cancer registries was similar, differences in the completeness of data and patients, which could have influenced the baseline characteristics and results, have to be considered. Baseline characteristics are of paramount importance for external validity of study results and should be studied carefully.^17^,^37^ Our findings may possibly be influenced by differences in the (under)registration of elderly patients with pancreatic cancer.^38^ On the other hand, age distribution was similar in the cancer registries. Furthermore, the number of included patients per cancer registry was similar to the expected number of patients based on the size of the cancer registry population, the incidence of pancreatic cancer, and the number of incidence years provided. The proportion of ‘unknown’ stages differed between the cancer registries. We hypothesized that this has only marginally influenced our results. The majority of patients with ‘unknown stage’ are likely to have stage III–IV disease and do not undergo further diagnostic procedures due to poor prognosis at the time of diagnosis. In addition, the distribution of ‘known’ stages was similar between the cancer registries. Second, the 7th edition, rather than the 8th edition, of the TNM classification was used in the analyses due to data availability. As shown by external validation studies, the 8th edition has more prognostic significance,^39^,^40^ but, on the other hand, was not yet in use during the study period (2012–2016). Third, this study included adjusted analyses for age group, but, nevertheless, residual confounding cannot be ruled out. Due to the low use of radiotherapy, adjusted analyses were not performed. In the sensitivity analyses, patients who deceased within 90 days after diagnosis or tumor resection were excluded and treatment strategies were re-investigated. In patients who did not undergo tumor resection, the influence of patients without pathological confirmation was also investigated. The sensitivity analyses showed that the original results were robust. Caution must be taken when drawing conclusions and indicating causal relations regarding the treatment strategies, since treatment selection bias cannot be ruled out.

To the best of our knowledge, this is the first study on elderly patients with stage I–II pancreatic cancer, in three European cancer registries, that gives insight into real-world data of treatment strategies and survival. These outcomes are relevant since the pancreatic cancer population is increasing in age and these patients are underrepresented in clinical trials.^7^,^41^ Future studies should focus on selection criteria for (non)surgical treatment so that clinicians can offer uniform and tailored treatment across countries and in (inter)national randomized trials. In this tailored treatment, quality of life plays a pivotal role and studies such as the Dutch Pancreatic Cancer Project (PACAP) will provide valuable data.^42^

## Conclusions

The treatment and survival of patients aged ≥ 70 years with stage I–II pancreatic cancer in the EURECCA Pancreas Consortium showed substantial variations between three European registries, including the rate of tumor resection, (neo)adjuvant chemotherapy, and palliative chemotherapy. The use of radiotherapy was limited. Survival of patients who did and did not undergo tumor resection also differed between the cancer registries. The findings of this study suggest that patients aged 70 years and older with stage I–II pancreatic cancer benefit from a higher tumor resection and chemotherapy administration rate.

## Electronic Supplementary Material

Below is the link to the electronic supplementary material.

Supplementary material 1 (DOCX 365 kb)

## Data Availability

The datasets used during this study are available from the corresponding author upon reasonable request.
