# Supplementary material for: Treatment and Survival of Elderly Patients with Stage I–II Pancreatic Cancer: A Report of the EURECCA Pancreas Consortium
Source: Ann Surg Oncol. 2020 May 9;27(13):5337–46. doi: 10.1245/s10434-020-08539-x (PMC7669775; doi:10.1245/s10434-020-08539-x)
Supplement: Supplementary file 1 — Supplementary material 1 (DOCX 365 kb) [file 10434_2020_8539_MOESM1_ESM.docx]

**SUPPLEMENTARY MATERIAL**

**Table S1. Description of cancer registries.**

|  | **Cancer registry** | | |
| --- | --- | --- | --- |
|  | **BE** | **NL** | **NOR** |
| **Registry** | Belgian Cancer Registry | Netherlands Cancer registry | Cancer Registry of Norway |
| **Organisation** | Population based | Population based | Population based |
|  |  |  |  |
| **Inhabitants (x10^6)** | 11 | 17 | 5 |
| **Incidence years in provided dataset** | 2012-2015 | 2012-2016 | 2012-2016 |
| **Coverage of data** | >98% | >95% | >98% |
| **Sources of data** | Pathology laboratories and use of medical claims data | Nationwide automated pathological archive (PALGA), National Registry of Hospital Discharge Diagnoses | Electronic reporting by physicians, reports from pathology laboratories, discharge and outpatient data, death registry |
| **Collection of survival data until** | 01-07-2018 | 31-01-2018 | 31-12-2017 |
| **Centralisation of surgery** | No | 18 hospitals | No |
|  |  |  |  |

**Table S2. Distribution of stages in registries.**

|  |  | **Cancer registry** | | | | | |
| --- | --- | --- | --- | --- | --- | --- | --- |
|  |  | **BE^a^** | | **NL^a^** | | **NOR** | |
|  |  | **N** | **%** | **N** | **%** | **N** | **%** |
| **Stage/Extent** | **IA** | 104 | 2.9 | 167 | 2.6 | Localised | |
|  | **IB** | 221 | 6.2 | 491 | 7.6 | 182 | 8.3 |
|  | **IIA** | 231 | 6.5 | 564 | 8.7 | Regional | |
|  | **IIB** | 513 | 14.4 | 792 | 12.3 | 465 | 21.1 |
|  | **III** | 273 | 7.6 | 781 | 12.1 | Distant | |
|  | **IV** | 1410 | 39.5 | 3392 | 52.6 | 1008 | 45.7 |
|  | **Unknown** | 822 | 23.0 | 264 | 4.1 | 551 | 25.0 |
| **^a^**Data from dynamic databases, numbers slightly differ from cohort included in study | | | | | | | |

**Table S3. Multivariable sensitivity analyses for overall survival, excluding patients who deceased within 90 days after diagnosis or tumor resection, including cancer registry, age group and chemotherapy as factors.**

|  |  | **Overall survival of patients who underwent tumor resection (N=1354)** | |  | **Overall survival of patients who did not undergo tumor resection (N=1243)** | |
| --- | --- | --- | --- | --- | --- | --- |
|  |  | **HR (95% CI)** | **P-value** |  | **HR (95% CI)** | **P-value** |
| **Cancer registry** | **BE** | **Reference** |  |  | **Reference** |  |
|  | **NL** | 1.10 (0.95-1.27) | 0.127 |  | 1.29 (1.11-1.49) | 0.001 |
|  | **NOR** | 0.70 (0.57-0.87) | 0.001 |  | 1.12 (0.93-1.35) | 0.217 |
| **Age group** | **70-74** | **Reference** |  |  | **Reference** |  |
|  | **75-79** | 1.19 (1.03-1.38) | 0.018 |  | 1.16 (0.97-1.39) | 0.099 |
|  | **≥80** | 1.20 (0.99-1.45) | 0.070 |  | 1.19 (1.00-1.40) | 0.040 |
| **(Neo)adjuvant chemotherapy^a^** | **No** | **Reference** |  |  | - | - |
|  | **Yes** | 0.82 (0.71-0.94) | 0.007 |  | - | - |
| **Palliative chemotherapy^b^** | **No** | - | - |  | **Reference** |  |
|  | **Yes** | - | - |  | 1.08 (0.92-1.27) | 0.332 |
| *HR: hazard ratio; CI: confidence interval* | | |  |  |  |  |
| ^a^Chemotherapy before or after tumor resection or both | | | |  |  |  |
| ^b^Chemotherapy in patients who did not undergo tumor resection | | | | | |  |

**Table S4. Sensitivity analyses for overall survival, excluding patients who deceased within 90 days after diagnosis or tumor resection, by cancer registry, age group and treatment strategy.**

|  | | | | | **Cancer registry** | | | | | | | | |
| --- | --- | --- | --- | --- | --- | --- | --- | --- | --- | --- | --- | --- | --- |
|  | | **Total** | | | **BE** | | | **NL** | | | **NOR** | | |
| **Age group** | **Treatment strategy** | **N** | **%** | **OS (95%CI)^a^** | **N** | **%** | **OS (95%CI)^a^** | **N** | **%** | **OS (95%CI)^a^** | **N** | **%** | **OS (95%CI)^a^** |
| **Total** | **Tumor resection + (neo)adjuvant chemotherapy** | 602 | 23.2 | 22 (19-25) | 271 | 33.7 | 21 (17-25) | 296 | 22.2 | 23 (19-27) | 35 | 7.6 | 27 (14-40) |
|  | **Tumor resection alone** | 752 | 28.9 | 18 (17-20) | 173 | 21.5 | 19 (15-24) | 360 | 27.0 | 15 (12-17) | 219 | 47.3 | 26 (23-28) |
|  | **Palliative chemotherapy** | 293 | 11.3 | 9 (8-11) | 173 | 21.5 | 11 (10-12) | 67 | 5.0 | 9 (8-11) | 53 | 11.4 | 9 (8-11) |
|  | **No treatment** | 951 | 36.6 | 8 (7-9) | 188 | 23.4 | 9 (7-11) | 607 | 45.6 | 8 (8-9) | 156 | 33.7 | 8 (7-9) |
|  | **Total** | **2599** | **100** | **13 (12-14)** | **805** | **100** | **15 (13-16)** | **1331** | **100** | **12 (11-12)** | **463** | **100** | **16 (14-19)** |
| **70-74** | **Tumor resection + (neo)adjuvant chemotherapy** | 366 | 41.6 | 24 (20-28) | 140 | 55.1 | 25 (19-31) | 198 | 44.6 | 24 (18-29) | 28 | 15.5 | 27 (9-45) |
|  | **Tumor resection alone** | 266 | 30.3 | 22 (18-26) | 36 | 14.2 | 26 (17-35) | 133 | 30.0 | 16 (13-19) | 97 | 53.6 | 34 (23-44) |
|  | **Palliative chemotherapy** | 118 | 13.4 | 11 (9-13) | 56 | 22.0 | 12 (9-14) | 36 | 8.1 | 11 (8-13) | 26 | 14.4 | 10 (8-11) |
|  | **No treatment** | 129 | 14.7 | 12 (10-13) | 22 | 8.7 | 9 (5-14) | 77 | 17.3 | 7 (6-8) | 30 | 16.6 | 12 (10-13) |
|  | **Total** | **879** | **100** | **18 (17-20)** | **254** | **100** | **18 (16-21)** | **444** | **100** | **16 (14-18)** | **181** | **100** | **25 (19-32)** |
| **75-79** | **Tumor resection + (neo)adjuvant chemotherapy** | 200 | 24.8 | 20 (18-23) | 104 | 40.5 | 20 (17-24) | 89 | 21.5 | 20 (15-26) | 7 | 5.2 | 27 (12-42) |
|  | **Tumor resection alone** | 298 | 37.0 | 16 (14-18) | 68 | 26.5 | 16 (10-22) | 149 | 36.0 | 13 (10-16) | 81 | 60.4 | 22 (17-27) |
|  | **Palliative chemotherapy** | 101 | 12.5 | 7 (2-12) | 53 | 20.6 | 11 (10-13) | 30 | 7.2 | 9 (7-11) | 18 | 13.4 | 7 (2-12) |
|  | **No treatment** | 205 | 25.5 | 8 (7-9) | 32 | 12.5 | 12 (10-14) | 145 | 35.0 | 8 (7-9) | 28 | 20.9 | 8 (6-9) |
|  | **Total** | **805** | **100** | **14 (12-15)** | **257** | **100** | **16 (13-19)** | **414** | **100** | **11 (10-13)** | **134** | **100** | **18 (14-21)** |
| **≥80** | **Tumor resection + (neo)adjuvant chemotherapy** | 36 | 3.9 | 21 (13-30) | 27 | 9.2 | 26 (9-21) | 9 | 1.9 | 20 (19-21) | 0 | 0.0 | - |
|  | **Tumor resection alone** | 188 | 20.5 | 17 (15-19) | 69 | 23.5 | 17 (11-24) | 78 | 16.5 | 16 (12-19) | 41 | 27.7 | 20 (11-29) |
|  | **Palliative chemotherapy** | 74 | 8.1 | 10 (8-11) | 64 | 21.8 | 10 (8-12) | 1 | 0.2 | 4 | 9 | 6.1 | 8 (7-10) |
|  | **No treatment** | 617 | 67.4 | 8 (7-9) | 134 | 45.6 | 8 (7-10) | 385 | 81.4 | 9 (8-9) | 98 | 66.2 | 8 (7-9) |
|  | **Total** | **915** | **100** | **10 (9-10)** | **294** | **100** | **11 (9-12)** | **473** | **100** | **9 (9-10)** | **148** | **100** | **10 (8-12)** |

^a^Median overall survival in months after tumor resection (patients who underwent tumor resection) or after diagnosis (patients who did not undergo tumor resection) and 95% confidence interval

**Figure S1 A-C. Overall survival of patients who underwent tumor resection by cancer registry for: (A) age group 70-74 years, (B) age group 75-79 years, (C) age group ≥80 years.**

**
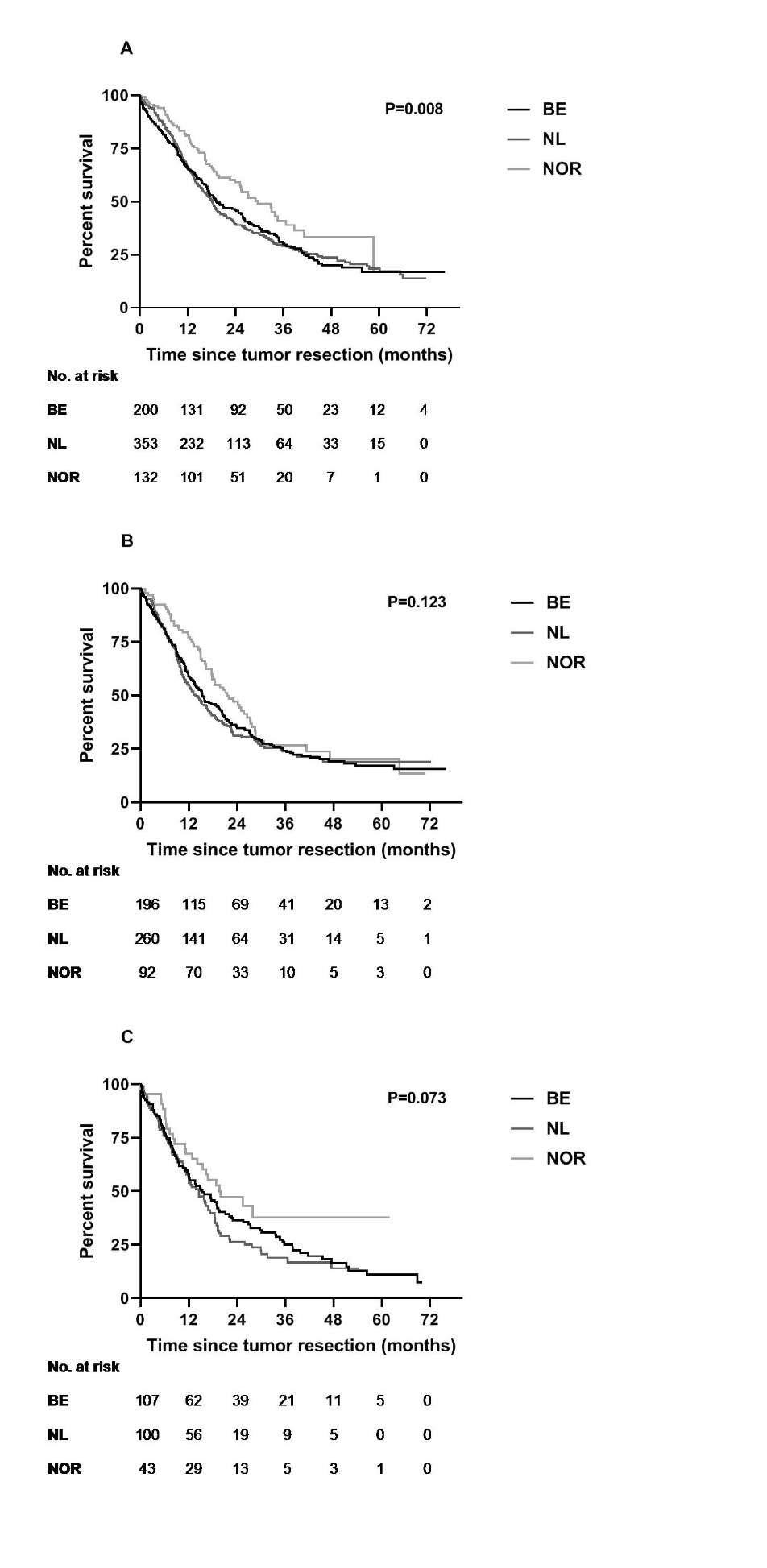
**

**Figure S2 A-C. Overall survival of patients who did not undergo tumor resection by cancer registry for: (A) age group 70-74 years, (B) age group 75-79 years, (C) age group ≥80 years.**

**
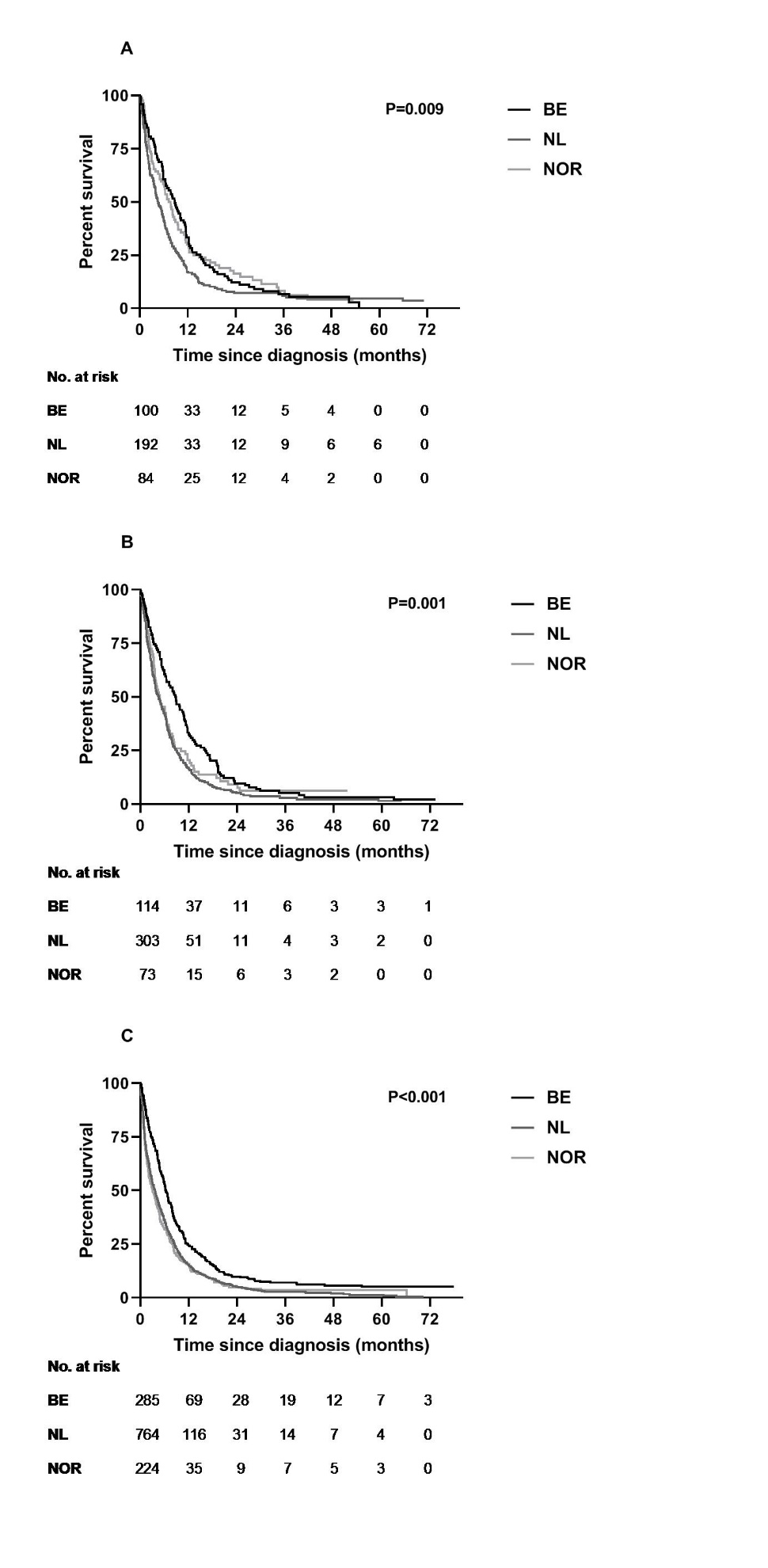
**
